# The effectiveness of assertive community treatment for elderly patients with severe mental illness: a randomized controlled trial

**DOI:** 10.1186/1471-244X-14-42

**Published:** 2014-02-15

**Authors:** Jolanda Stobbe, André I Wierdsma, Rob M Kok, Hans Kroon, Bert-Jan Roosenschoon, Marja Depla, Cornelis L Mulder

**Affiliations:** 1 Department of Psychiatry, Epidemiological and Social Psychiatric Research institute, Erasmus MC, PO Box 2040 Dp-0122, Rotterdam, CA 3000, The Netherlands; 2 Parnassia Psychiatric Institute, department BavoEuropoort, Centre for Mental Health Care, Prins Constantijnweg, Rotterdam, The Netherlands; 3 Parnassia Psychiatric Institute, department Parnassia, Centre for Mental Health Care, Monsterweg, The Hague, The Netherlands; 4 Trimbos Institute, Netherlands Institute of Mental Health and Addiction, Utrecht, Da Costakade, The Netherlands; 5 Department of General Practice & Elderly Care Medicine, EMGO Institute for Health and Care Research, VU University Medical Centre, Van der Boechorsstraat, Amsterdam, The Netherlands

**Keywords:** Assertive community treatment, Severe mental illness, Dropout, Elderly, HoNOS65+, RCT

## Abstract

**Background:**

Due to fragmented mental, somatic, and social healthcare services, it can be hard to engage into care older patients with severe mental illness (SMI). In adult mental health care, assertive community treatment (ACT) is an organizational model of care for treating patients with SMI who are difficult to engage. So far all outcome studies of assertive community treatment have been conducted in adults.

**Methods:**

In a randomized controlled trial design we compared the effectiveness of ACT for elderly patients with that of treatment as usual (TAU). Sixty-two outpatients (60 years and older) with SMI who were difficult to engage in psychiatric treatment were randomly assigned to the intervention or control group (32 to ACT for elderly patients and 30 to TAU). Primary outcomes included number of patients who had a first treatment contact within 3 months, the number of dropouts (i.e. those discharged from care due to refusing care or those who unintentionally lost contact with the service over a period of at least 3 months); and patients’ psychosocial functioning (HoNOS65+ scores) during 18 months follow-up. Secondary outcomes included the number of unmet needs and mental health care use. Analyses were based on intention-to-treat.

**Results:**

Of the 62 patients who were randomized, 26 were lost to follow-up (10 patients in ACT for elderly patients and 16 in TAU). Relative to patients with TAU, more patients allocated to ACT had a first contact within three months (96.9 versus 66.7%; X^2^ (df = 1) = 9.68, p = 0.002). ACT for elderly patients also had fewer dropouts from treatment (18.8% of assertive community treatment for elderly patients versus 50% of TAU patients; X^2^ (df = 1) = 6.75, p = 0.009). There were no differences in the other primary and secondary outcome variables.

**Conclusions:**

These findings suggest that ACT for elderly patients with SMI engaged patients in treatment more successfully.

**Trial registration:**

NTR1620

## Background

Due to fragmented mental, somatic, and social healthcare services, it can be hard to engage into care older patients with severe mental illness (SMI) who have problems in multiple life domains and problems in treatment motivation [1-4]. Assertive community treatment (ACT) was developed as an integrated model to meet the needs of difficult-to-engage patients with complex problems [5]. Critical components of ACT associated with reducing hospital admissions were shared caseload, community based services, 7x 24 hour services, a team leader who participated in patient care, full responsibility for treatment services, daily team meetings and time unlimited services [6]. Although there is no agreement on which critical components of ACT are associated with psychosocial outcomes, better outcomes have both been shown to be associated with having a better team structure and with having a consumer provider in the team [7-9]. Better engagement was associated with a smaller caseload in ACT than in TAU, and with a shared caseload [10].

In the United States, ACT reduced hospital admissions more than treatment as usual (TAU) [5,11]. European studies however showed mixed effects of ACT when compared with TAU, due possibly to better quality of care in the TAU control-conditions and/or inadequate implementation of key ACT-components. Reduced effectiveness of ACT in Europe could also be explained by a loss of focus on preventing admissions when ACT-teams are confronted with very strict admission criteria, or conversely, when in-patient beds are readily available [12-15].

One group of patients who might benefit from ACT are elderly patients with SMI who are difficult to engage, have heterogeneous care needs, such as psychiatric as well as somatic problems, and have problems with activities of daily living, housing and social support [16,17]. Only one study has focused on ACT for the elderly (ACTE). Although results suggested that the ACT model supplemented with specific care for the elderly was suitable for this subgroup, the study presented no data on the effectiveness of this service [18].

To meet the different needs of older patients with SMI who are difficult to engage, a specialized ACTE team was started. We tested its effectiveness in a randomized controlled trial (RCT). We hypothesised that ACTE, as compared to Treatment As Usual (TAU), more often succeeds in establishing contact with patients within 3 months of their signing up for care, has fewer drop outs, and has better effects on patients’ psychosocial functioning (primary outcome variables). We also hypothesized that ACTE would meet patients’ unmet needs more effectively, and would reduce mental healthcare use (secondary outcome variables) as compared to treatment as usual.

## Methods

### Intervention

ACT is a community-based treatment approach for outpatients whose SMI results in difficulties in daily living activities and social functioning often including problems with relationships, physical health, addiction, work, daytime activities, and living conditions. ACT was developed for patients who are high users of inpatient hospital services and who are unwilling to use mental health services [19]. Unlike other community-based programs, ACT provides individualized services directly to the patients. To meet patients’ various needs, a multidisciplinary team provides psychiatric, somatic and rehabilitation treatment. Team members are trained in the areas of psychiatry, social work, nursing, substance abuse, and rehabilitation [20]. Key features of ACT are: assertive engagement, a small caseload (maximum of 10 patients per clinician); shared caseload (i.e. all clinicians collaborated closely on each patient using one treatment plan); and community-based and assertive services on a time-unlimited basis [5,19]. ACTE was implemented using the ACT manual developed for adults. The descriptions in this manual include the team approach and the duties of each discipline [19]. The ACTE team was staffed by: a substance-abuse specialist, a rehabilitation worker, a social worker, a psychiatric nurse, a nurse specialized in somatic care, a community mental health nurse and a psychiatrist (the last two were both specialized in treating elderly people).

Treatment as usual was provided by three community mental health teams for elderly patients. Two of these teams were for patients with primary psychiatric disorders, and one was for patients with cognitive disorders. The teams provided regular mental health services, including psychiatric care on an outreach basis. Various disciplines (including community mental health nurses, a psychiatrist, and a psychologist) were individually responsible for the patients and their treatment plans (no shared caseload); their caseload was relatively high (more than 25 patients per practitioner). All clinicians were specialized in treating elderly people.

### Trial design and recruitment

The study was carried out by Parnassia Psychiatric Institute, department BavoEuropoort, a mental healthcare centre in the greater Rotterdam area in the Netherlands. BavoEuropoort provides inpatient and outpatient care in an urban population of some 1.3 million people.

The ACTE study was designed as a parallel group randomized controlled trial, with one intervention group and one control group. The study was approved by the Dutch Union of Medical-Ethical Trial Committees for mental health organizations. See Stobbe et al. [21] for a more detailed description of the study.

All elderly patients referred to the BavoEuropoort mental healthcare centre in Rotterdam (the Netherlands) between July 2008 and July 2010 were screened with regard to meeting the inclusion criteria.

General practitioners or municipal health services referred patients according to usual procedures (the person who referred the patient filled out a form describing the patient’s characteristics and current problems). Inclusion and exclusion criteria were filled in and checked by the clinicians of the ACTE and the community mental health teams. The initial inclusion criteria included: 1.) age 65 years or older, 2.) presumption of a severe mental illness (for example schizophrenia spectrum disorders or major affective disorders); 3.) problems in four or more of the following areas: daily functioning, (e.g. personal hygiene, social relationships), daytime activities, addiction, financial problems, housing, somatic problems, or police contacts; and 4.) difficulties in engaging in treatment (for example patients who were unwilling to use mental health services, or those who had a history of involuntary admissions or of drop-out from mental healthcare). There was one exclusion criterion: the presumption of moderate to severe cognitive impairment [21].

Because the inclusion rate was low during the first year of inclusion (n = 35), we extended the inclusion period to a total of 24 months. Extending the inclusion period was not sufficient for recruitment, and therefore the inclusion criteria were broadened: after 1 year we broadened the inclusion criteria by lowering the minimum age to 60 years. We also dropped problems in various domains as an inclusion criterion, since patients often received no medical or psychiatric treatment so that only limited information was available when patients were referred.

We compared patients who were recruited during the first year of the study to patients who were recruited later. Patients’ level of unmet needs and psychosocial functioning at baseline did not differ between patients included during the first year of the study and patients included after we broadened the inclusion criteria. Because we lowered the minimum age after 1 year, patients included after this year were younger than patients included the first year of the study (72.1 years, standard deviation (SD) 8.7 versus 76.7 years (SD 7.2). T-test 2.24, degrees of freedom (df) = 60, p = 0.029).

### Randomization

To allocate the patients to ACTE or TAU we used a randomization list generated by a computer (http://www.randomizer.org). Participants who fulfilled the inclusion criteria received a number chronologically from the ACTE service administrator. On the basis of this number, an opaque sealed envelope with the corresponding number was opened and the patient was allocated randomly.

### Outcomes

The study focused on three primary outcome measures. First we computed the number of patients who had a first treatment contact with the mental healthcare worker within 3 months of being signed up for care. Second, the number of dropouts during the follow-up period was assessed where there were two categories: being discharged for refusing care and (temporary) unintentional loss of contact with the service for at least 3 months; and thirdly psychosocial problems over time.

Secondary outcome measures comprised the number of unmet needs over time, the number of in-patient psychiatric hospital admissions, duration of admission and the number of crisis contacts [21].

Outcomes were assessed at three time points: at baseline (T1), at 9 months after baseline (T2), and 18 months after baseline (T3).

### Instruments

Demographic characteristics and dropout data were collected from patients’ electronic files. The number of psychiatric hospital admissions, duration of admission and the number of crisis contacts were collected from the central system of registration.

The Dutch version of the Health of the Nation Outcome Scales for elderly people (HoNOS65+) was used to assess the severity of psychosocial problems [22,23]. The HoNOS65+ is divided into 12 items: overactive and aggressive behaviour, non-accidental self-injury, problem drinking or drug-taking, cognitive problems, physical illness or disability problems, hallucinations and delusions, depressed mood, other mental and behaviour problems, problems with relationships, problems with activities of daily living, problems with living conditions, and problems with occupation and activities. We used the sum score of the HoNOS65+. All items were scored on a 5-point scale, from 0 (no problem) through 1 (minor problem), to 2 (mild problem), 3 (moderate problem), and 4 (severe problem). The Dutch version of the HoNOS65+ has been shown sensitive enough to measure change [23,24].

The short Dutch version of the Camberwell Assessment of Needs for the Elderly (CANE, staff member version) [25,26] was used to measure care needs in twenty-four areas. Each item was scored as 0 = no need, 1 = met need (due to an intervention) or 2 = unmet need (intervention is needed, or intervention had no effect). We used the sum score of the unmet needs. The validity and reliability of the original scale were good [25] and acceptable for the Dutch version [26].

Measuring model fidelity of ACT is a method to evaluate how close ACT was implemented to the academic ideal. The model fidelity of ACTE and TAU was measured two years after the start of the ACTE team, using the Dutch version of the Dartmouth Assertive Community Treatment Scale (DACTS) [27,28]. The scale items were divided into three sections: human resources, organizational boundaries and nature of services.

### Data-collection procedure

Outcome data were collected by psychology students on the basis of a face-to-face contact with each patient, which was combined with information provided by the clinician and with data in the patients’ electronic files. If patients refused contact, data were collected only on the basis of information provided by the clinician and of data in the patients’ electronic files. Data were analysed anonymously. For practical issues, raters could not be blinded for the treatment condition. Because we were dealing with unmotivated patients, the instruments were assessed from the raters’ point of view.

### Statistical analyses

First, all variables were checked for outliers and missing values. Next, we compared socio-demographic and clinical characteristics at baseline. Chi-square tests were used to analyze differences between the intervention and control group in the number of patients who had a first treatment contact within three months and the number of dropouts. To test differences regarding psychosocial functioning (total score HoNOS65+), the intervention and control group were compared using regression analysis, with centred psychosocial functioning at baseline as covariate and treatment condition as factor. Finally, the Wilcoxon test was used to investigate secondary outcomes, including within-group changes in unmet needs, the number of hospital days and crisis contacts during follow-up. The statistical significance level was set at p < 0.05. The Statistical Package of the Social Sciences (SPSS), version 17 for Windows, was used for all analyses.

The research question was to demonstrate differences between ACTE and TAU. In order to avoid overestimation, data were analyzed on an intention-to-treat basis. This meant that patients who did not fully adhere to the protocol or patients who received the treatment from the group they were not allocated to, were kept in their original group for the analyses [29].

## Results

### Model fidelity

Table 1 shows that the ACTE team had a mean DACTS score of 3.6, meaning that the ACT model was implemented to a moderate degree [28,30]. The TAU teams had lower model fidelity scores (all 3 teams had a score of 2.4). ACTE scored high on the following components of ACT: the small and shared caseload, and time-unlimited services [31,32]. While ACTE had the maximum scores for the other components of ACT (community based services and assertive outreach), almost all TAU teams also scored high on these components. The ACTE and the TAU teams also had high scores on components such as explicit admission criteria, a team leader who participated in patient care, and responsibility for hospital admission. ACTE had low model fidelity on the following components: a vocational specialist and consumer provider in the team, the frequency of contact, the intensity of service, the intake rate, dual-disorders treatment groups, work with support system, and responsibility for crisis services.

**Table 1:** Difference in DACTS score between ACTE and TAU

**DACTS criteria**	**PG team**	**GP team North**	**GP team South**	**ACTE**
Small caseload	1	1	2	4
Team approach	1	2	1	5
Program meeting	1	1	1	5
Practicing team leader	5	5	5	5
Continuity of staffing	5	5	2	4
Staff capacity	5	5	2	5
Psychiatrist on staff	1	1	2	3
Nurse on staff	3	3	3	5
Substance abuse specialist on staff	1	1	1	3
Vocational specialist on staff	1	1	1	2
Program size*	4	5	3	3
**Team structure mean:**	**2.5**	**2.7**	**2.1**	**4**
Explicit admission criteria	4	4	5	4
Intake rate	1	1	1	1
Full responsibility for treatment services	2	3	4	5
Responsibility for crisis services	1	1	1	1
Responsibility for hospital admissions	4	4	5	5
Responsibility for hospital discharge planning	1	2	3	5
Time-unlimited services	1	4	2	5
**Organizational boundaries mean:**	**2,0**	**2,7**	**3**	**3,7**
In-vivo services	5	4	2	5
No drop-out policy	5	4	3	5
Assertive engagement mechanisms	5	3	5	5
Intensity of service	1	1	1	2
Frequency of contact	1	1	1	1
Work with support system	5	1	5	2
Individualized substance abuse treatment	1	1	1	4
Dual disorder treatment groups	1	1	1	1
Dual disorders model	1	1	1	3
Role of consumers on treatment team	1	1	1	1
**Nature of services mean:**	**2,6**	**1,9**	**2,1**	**2,9**
TOTAL MEAN SCORE DACTS*	**2,4**	**2,4**	**2,4**	**3,6**

Each item is rated on a 5-point scale from 1 (not implemented) to 5 (fully implemented), the scale items of the DACTS are divided into three sections: human resources, organizational boundaries and nature of services (bold data are mean scores of these sections).

*Program size is not included in score summary.

0–2.9: inadequate implementation ACT model.

3.0-4.1: moderate implementation ACT model.

4.2-5.0: full implementation ACT model.

**Figure 1:**
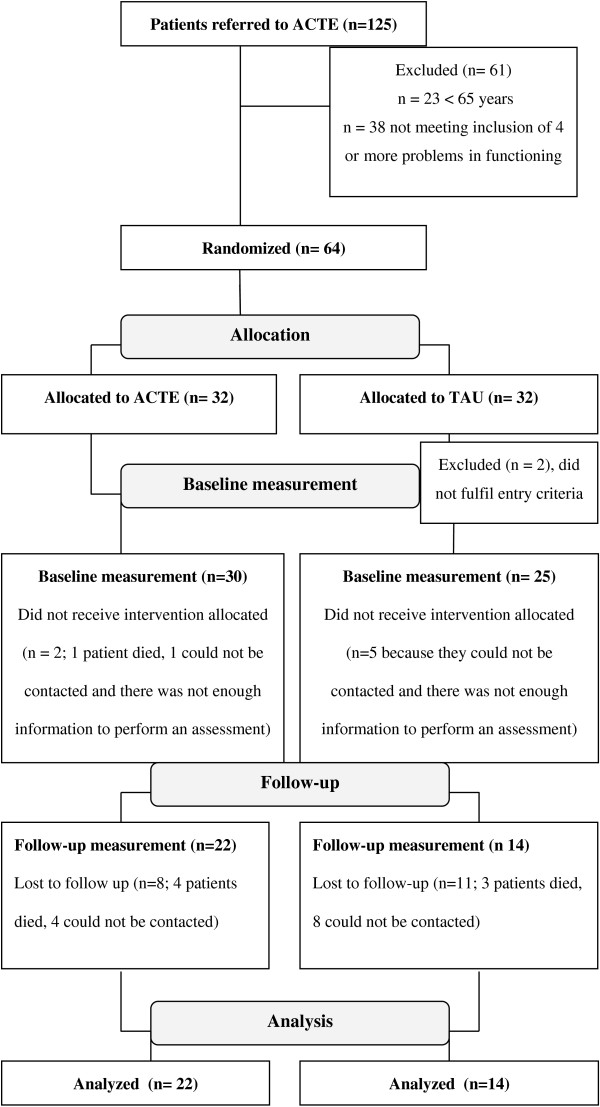
CONSORT flow of participants through the study.

The flowchart of the study is presented in Figure 1. Sixty-one patients of the 125 patients referred did not fulfill the inclusion criteria, and were excluded. Twenty-three patients were excluded because they were younger than 65 years and thirty-eight patients were excluded because they did not meet the narrow inclusion criteria at the start of the study, of four or more problems in daily living activities and functioning. At the end of the inclusion period we recruited 64 participants. Directly after randomization two patients, allocated to TAU, were excluded from care, these patients did not fulfill the entry criteria (incorrect entry of the patient into the trial).

Two patients who had originally been allocated to TAU received ACTE. These patients were analysed in TAU, according to the intention to treat principle. Patients were lost to follow-up because they were deceased or could not be contacted – sometimes because they were admitted to in-patient services (elderly homes) but mostly because patients didn’t open the door or refused contact. At follow-up, assessments were available of 22 patients in ACTE and 14 patients in TAU (see Figure 1). The mean time between these follow-up measurements was 17.6 months for ACTE patients (SD 5.2) versus 17.8 months (SD 5.7) for patients in TAU. Due to loss to follow-up, we used only two measurements per patient. For patients with three measurements (n = 25) we used only the first and last measurement. There were 7 patients with two measurements. Thus, for these we used both measurements.

### Baseline characteristics

Demographic details, service attendance, HoNOS65+ total score and, total unmet needs are shown in Table 2. At baseline, there were no differences in demographic characteristics between patients receiving ACTE or patients receiving TAU.

**Table 2:** Characteristics of the study population

**Characteristics**	**ACTE n**=**32**	**TAU n**=**30**
**Mean age (SD)**	74.4 (7.0)	75.1 (9.3)
**Mean age at first contact with mental health services (SD)**	61.5 (16.5)	60.7 (21.5)
**Gender (%)**		
Male	16 (50)	10 (33.3)
Female	16 (50)	20 (66.7)
**Marital status (%)**		
Unmarried	14 (43.8)	9 (30.0)
Married	4 (12.5)	2 (6.7)
Divorced	7 (21.9)	10 (33.3)
Widowed	7 (21.9)	9 (30.0)
**Living situation (%)**		
Independent	27 (84.4)	27 (90.0)
Other	5 (15.7)	3 (10.0)
**Country of birth (%)**		
The Netherlands	29 (90.6)	22 (73.3)
Other	3 (9.4)	8 (26.7)
**Diagnosis axis I (%)**		
Schizophrenia spectrum disorders	11 (34.4)	11 (36.7)
Mood disorder	5 (15.6)	3 (10.0)
Cognitive impairment	4 (12.5)	7 (23.3)
Other disorders	12 (37.5)	9 (30.0)
**Psychiatric admission (%)**		
Yes	10 (31.3)	7 (23.3)
No	22 (68.8)	23 (76.7)
Total HoNOS65+ score mean (SD)*	21.17 (3.87)	20.4 (4.58)
**Total unmet needs score**	7.5 (1-15)	6.5 (2-13)
Median (range)*		

*Based on 30 ACTE patients and 25 CAU patients.

### First contact within 3 months and dropout patients

First contact and the dropout data are presented in Table 3. Within three months of signing up for care, patients allocated to ACTE had contact with mental healthcare workers significantly more often than those allocated to TAU. While ACTE also had significantly fewer dropouts from treatment than TAU did, within the TAU condition two patients were lost only temporarily (for 8 and 10 months).

**Table 3:** **First contact and dropout** (**during follow**-**up**) **stratified by treatment programme**

	**ACTE n = 32 (%)**	**TAU n = 30 (%)**	**Test of significance**
**First contact within 3 months**			
Yes	31 (96.9)	20 (66.7)	X^2^ (df = 1) = 9.68
No	1 (3.1)	10 (33.3)	p = 0.002
**Drop-out**			
Yes	6 (18.8)	15* (50)	X^2^ (df = 1) = 6.75
No	26 (81.3)	15 (50)	p = 0.009

chi-squared test was used.

*2 patients dropped out temporarily (for 8 and 10 months).

### Psychosocial functioning

The mean total HoNOS65+ scores at follow-up showed an improvement in psychosocial functioning in both groups. At baseline, the mean score in the ACTE group was 20.6 (SD 4.1); at follow-up it was 16.0 (SD 5.0), t-test: t = 3.71 (df = 21), CI: 2.0-7.2, p = 0.001. The effect range lay between a 5-point increase in psychosocial problems and a 15-point decrease. The mean baseline score for TAU patients was 20.4 (SD 5.3) and at follow-up it was 16.1 (SD 6.7), t-test: t = 1.86 (df = 13), CI = −.68-9.3, p = 0.085. The effect range lay between + 9 and −19. Table 4 shows the result of the regression analysis, which indicates that no significant effect was found for treatment condition on psychosocial outcome.

**Table 4:** **Regression analysis of a comparison of psychosocial functioning** (**HoNOS65**+ **follow**-**up**) **and random treatment condition**, **analysis of covariance**

**Source**	**B (SE)**	**95% CI**	**p-value**
**Intercept**	16.1 (1.54)	12.96 −19.22	0.000
**Baseline measurement**	0.08 (0.22)	−.36 −0.53	0.699
**Treatment condition**	0.10 (1.97)	−4.10 −3.91	0.962

SE = standard error; CI = confidence interval.

### Unmet needs

In both conditions, the total median number of unmet needs decreased significantly over time. At baseline, the median total unmet needs score for ACTE patients was 7.5 (range 1–15); at follow-up it was 2.5 (range 0–6, Wilcoxon W test z = −3.50, p = < 0.001). At baseline, TAU patients had a median score of 8 (range 3–13) and at follow-up it was 2 (range 0–11), respectively (Wilcoxon W test z = −3.11, p = 0.002).

### Hospital days and crisis contacts

Three patients were hospitalized 2 years before the start of the study (2 patients in ACTE and 1 in TAU). After the start of the study, 4 patients in both conditions were hospitalized. Seven patients in ACTE and no patients in TAU had a crisis contact before the start of the study. Two years after the start of the intervention, 5 patients who received ACTE and 4 patients in TAU had had a crisis contact. As very few patients had been admitted or had had crisis contacts, these variables were not analyzed statistically.

## Discussion

The major findings of this study were that ACTE succeeded better than TAU in engaging patients into care within 3 months, and was also able to prevent dropout from treatment (18.8% dropout in ACTE patients versus 50% in the TAU condition). Our results also demonstrated that ACTE did not produce better outcomes with respect to psychosocial functioning, unmet needs or mental health care use.

By showing better engagement but no effects on psychosocial functioning, these results are in line with studies on the effects of ACT in adults [33-35]. Some studies explained the lack of added value of ACT due to the high quality of TAU teams [12,14,36] or due to a lack of implementation of evidence based modules in ACT teams [33]. In their study, Killaspy et al. explained that better engagement in ACT was associated with a smaller caseload in ACT than in TAU, and with the team approach (shared caseload) [10]. These characteristics were also present in our own ACTE condition. TAU was characterized by larger caseloads and individual case management. It may be that having a large caseload as well as individual case management both limit the possibilities for trying to make contact with difficult-to-engage patients and for preventing dropout.

Although there is no agreement on which critical components of ACT are associated with psychosocial outcomes in elderly patients, better outcomes in adults have been shown to be associated both with having a better team structure (shared caseload, daily team meetings, and a team leader who participated in patient care) and with having a consumer provider in the team [7-9]. Both ACTE and TAU did not have a consumer in the team whereas also in both conditions the team leader participated in care, thereby limiting the differences between ACTE and TAU.

Various reasons are possible for the lack of differences with regard to outcome in psychosocial functioning. First, given the differences in the numbers of patients who dropped out of care, it is possible that patients who dropped out of TAU had worse psychosocial outcomes, which led to a selection bias in TAU. Second, ACTE may have caused selection bias by preventing the dropout of patients who had worse prognoses than the others. Third, TAU used components of ACT, so in some critical components of ACT differences between the intervention and control group were small. Also contact frequency in ACTE was low. Lack of integrated care and degree of contacts was associated with lack of differences found in effect studies in Europe [37]. Various studies have shown that ACT has the best results when it is implemented in full accordance with the original ACT model [38-40]. However, other studies showed no association with model fidelity and outcome in ACT [10,34,41]. In the present study, ACTE had moderate model fidelity: for example ACTE did not fully implement a consumer in their team and the contact frequency was low. Also ACTE did not include a psychologist in the team [41,42]. This may have limited its effectiveness. Nevertheless it could be that, as in line with earlier mentioned studies, ACTE does not have added value in improving psychosocial outcomes [34,41].

### Strengths and limitations

The strength of this study is that we managed to include difficult-to-engage patients with SMI in an RCT. To our knowledge this study is the first RCT that investigated the effectiveness of a special ACT team for elderly patients. One limitation is the low number of patients enrolled in this study and the high number of patients lost to follow-up, which meant that the power to detect changes was low (lack of power resulting in a type 2 error). Another limitation is the selection bias; the findings do not apply to all elderly patients with SMI since data were collected in one institution in an urban setting and most patients were, at baseline, difficult to engage. Furthermore it is possible that differences in psychosocial functioning between the intervention and control group were not detected because of measurement limitations. The sum score of the HoNOS65+ has been criticized for not properly measuring change in psychosocial functioning [43]. Finally not all assessments were filled out after a face-to-face contact with the patient and raters were not blind for the treatment condition.

## Conclusions

In conclusion, ACTE had better results than TAU with regard to engaging patients into treatment and fewer dropouts. However, we could not demonstrate that ACTE led to better psychosocial functioning. To replicate these findings further research is needed in a larger group of patients.

## Abbreviations

ACT: Assertive community treatment; ACTE: Assertive community treatment for the elderly; CANE: Camberwell assessment of needs elderly; CI: Confidence interval; DACTS: Dartmouth assertive community treatment scale; df: degrees of freedom; HoNOS: Health of the Nation Outcome Scale; RCT: Randomized controlled trial; SD: Standard deviation; SMI: Severe mental illness; SPSS: Statistical package of the social science; TAU: Treatment as usual.

## Competing interests

JS, CLM, RMK and BR are employees of Parnassia Psychiatric Institute. A department of this institute funded the study. All authors declare that they have no conflicts of interests and none of the authors received payments as a consequence of authorship for this manuscript.

## Authors’ contributions

JS and CLM constructed the design of the study. CLM, HK, BR and MD participated in its design. AIW was consulted in the study methodology and the statistical approach. JS carried out (under supervision of AIW) the statistical analysis and wrote this article. CLM and AIW assisted in drafting the manuscript. HK, RMK, BR and MD made revisions to the manuscript. All authors read and approved the final manuscript.

## Pre-publication history

The pre-publication history for this paper can be accessed here:

http://www.biomedcentral.com/1471-244X/14/42/prepub
